# Streamlined selection of cancer antigens for vaccine development through integrative multi-omics and high-content cell imaging

**DOI:** 10.1038/s41598-020-62244-z

**Published:** 2020-04-03

**Authors:** Ki-Cheol Han, Daechan Park, Shinyeong Ju, Young Eun Lee, Sun-Hee Heo, Young-Ae Kim, Ji Eun Lee, Yuna Lee, Kyong Hwa Park, Se-Ho Park, Hee Jin Lee, Cheolju Lee, Mihue Jang

**Affiliations:** 1 Center for Theragnosis, Biomedical Research Institute, Korea Institute of Science and Technology, Seongbuk-Gu, Seoul, 02792 Republic of Korea; 2 Department of Biological Sciences, College of Natural Sciences, Ajou University, Suwon, 16499 Republic of Korea; 3 College of Life Science and Biotechnology, Korea University, 145 Anam-ro, Seongbuk-gu, Seoul, 02841 Republic of Korea; 4 Department of Pathology, Asan Medical Center, University of Ulsan College of Medicine, 88 Olympic-ro 43-gil, Songpa-gu, Seoul, 05505 Republic of Korea; 5 Asan Center for Cancer Genome Discovery, Asan Institute for Life Sciences, University of Ulsan College of Medicine, Asan Medical Center, Seoul, 05505 Republic of Korea; 6 Oncology/Hematology, Department of Internal medicine, Korea University College of medicine, Seongbuk-Gu, Seoul, 02841 Republic of Korea; 7 Department of Converging Science and Technology, KHU-KIST, Kyung Hee University, Seoul, 02447 Republic of Korea

**Keywords:** Bioinformatics, High-throughput screening, Immunological techniques, Proteomic analysis, Cancer, Biochemistry, Biological techniques, Cancer, Computational biology and bioinformatics, Immunology

## Abstract

Identification of tumor antigens that induce cytotoxic T lymphocytes (CTLs) is crucial for cancer-vaccine development. Despite their predictive ability, current algorithmic approaches and human leukocyte antigen (HLA)-peptidomic analysis allow limited selectivity. Here, we optimized a method to rapidly screen and identify highly immunogenic epitopes that trigger CTL responses. We used a combined application of this method involving immune-specific signature analysis and HLA-associated peptidomics using samples from six patients with triple-negative breast cancer (TNBC) in order to select immunogenic HLA epitopes for *in vitro* testing. Additionally, we applied high-throughput imaging at the single-cell level in order to confirm the immunoreactivity of the selected peptides. The results indicated that this method enabled identification of promising CTL peptides capable of inducing antitumor immunity. This platform combining high-resolution computational analysis, HLA-peptidomics, and high-throughput immunogenicity testing allowed rapid and robust identification of highly immunogenic epitopes and represents a powerful technique for cancer-vaccine development.

## Introduction

Cancer immunotherapy to boost T cell-mediated immune response in order to target and eliminate cancer cells has proven therapeutically efficacious in a variety of human malignancies^1^. In particular, therapeutic cancer vaccines against tumor-related epitopes that directly stimulate T cells have been clinically effective and are currently available^2^. Cancer cells express several antigens, including self-antigens derived from tumor tissues, as well as mutation-derived antigens (i.e., neoantigens), that can be recognized as foreign antigens by the host immune system^3–5^. Recently, cancer vaccines targeting individual neoantigens have become an attractive form of cancer therapeutics by virtue of their ability to elicit a robust T cell immune response^6–8^. Three independent clinical studies demonstrated the efficacy of this approach in eliciting neoantigen-specific T cell responses in patients with late-stage melanoma^9–11^. Nevertheless, several challenges to personalized cancer treatment targeting distinct neoantigens remain as obstacles to wide clinical application of these vaccines. First, the prediction of highly immunogenic neoantigens is difficult. Recent advances in next-generation sequencing technology and MHC-epitope databases allow the accurate prediction of neoantigens; however, existing tools are inadequate for accurate prediction of immunogenicity due to the multiple associated factors, such as proteasomal-processing ability, intracellular routing, specific binding to highly polymorphic HLA molecules, and peptide/MHC (p/MHC)-complex stability^6,12^. Therefore, it is necessary to optimize the predictive accuracy of MHC-binding affinity and selection of immunogenic neoantigens in order to improve currently available algorithms and bioinformatics tools^13^.

Traditional cancer vaccines target different types of self-antigens, including overexpressed tumor-specific proteins and oncogenes that might also represent universal antigens among patients with the same malignancy^14–16^. Self-antigens derived from tumor-related proteins are grouped into three categories^17,18^: tumor-associated antigens (TAAs), tumor-specific antigens (TSAs), and cancer/testis antigens (CTAs). TAAs are typically overexpressed in tumor tissues relative to their levels in other tissues, whereas TSAs are exclusively expressed in cancer cells, and CTAs are expressed only in germline tissues and trophoblastic cells in addition to being expressed in cancer cells. Using genetic and immunological approaches, several self-antigens have been identified as adequate targets for cancer immunotherapy^16^; however, vaccination against self-antigens can still potentially induce central- and peripheral-tolerance responses resulting in low therapeutic efficacy^5,19^ or an autoimmune response against normal tissues^6^. Therefore, identification of appropriate self-antigens is important for successful cancer-vaccine immunotherapy.

**Figure 1:**
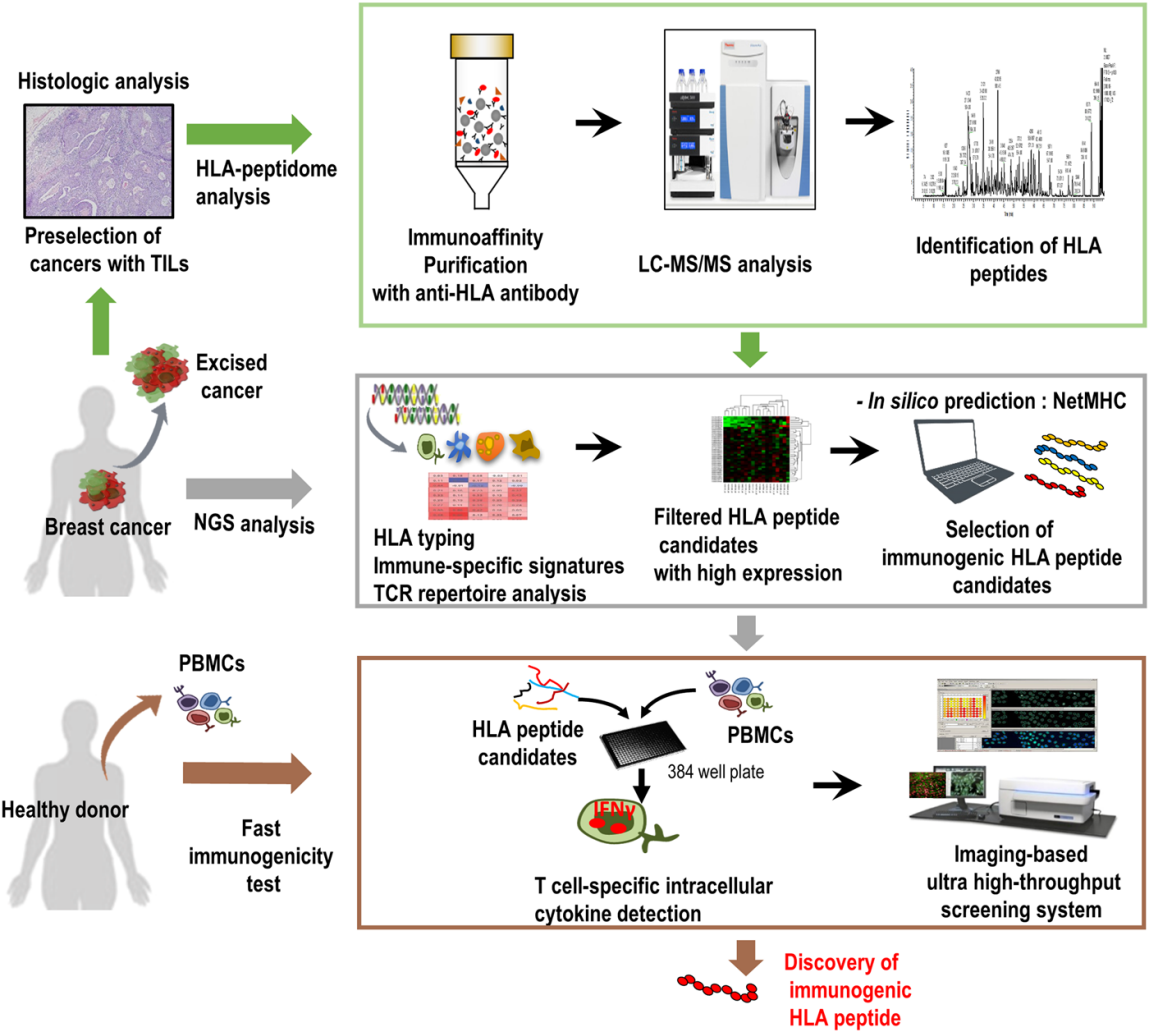
Scheme of the rapid high-throughput approach for discovering natural CTL epitopes. Preselected TIL-resident TNBC tumors underwent HLA-peptidomic analysis to identify HLA-bound peptides. Integrated WTS data revealed a higher priority to select promising HLA-peptides *via* high-resolution bioinformatics analysis, showing immune-cell-specific signatures and TCR-repertoire diversity in tumors. Combined NGS analysis and the use of predictive algorithms for MHC-binding affinity enabled selection of highly immunogenic HLA-peptide candidates. Analysis of IFNγ-producing CD8^+^ T cell response using the high-content imaging system in a 384-well format at the single-cell level for discovery of immunogenic HLA epitopes eliciting a CTL response.

Cancer vaccines targeting cancer antigens have been developed using multiple approaches, including peptides, proteins, nucleic acids, and viral vectors^20^. Among these strategies, peptides used in cancer vaccines are 20–30 amino acids, showing lower antigen complexity and low costs for manufacturing^21^. In particular, MHC class-І-restricted peptides (i.e., CTL epitopes) recognized by CD8^+^ CTLs play a key role in attacking cancer cells by promoting the activation and proliferation of antigen-specific CTLs^22^. To develop effective CTL-associated peptide vaccines, many methods use bioinformatics algorithms and proteomics approaches to identify multiple HLA-class-bound peptides^23,24^. Recently, proteomics approaches associated with mass spectrometry (MS)^25^ and specifically LC-MS/MS, were used for HLA-peptidomic analysis to directly facilitate the discovery of numerous natural peptides in complex with specific HLA molecules expressed on cell surfaces^26,27^. Additionally, recent advances in MS-based technologies led to the development of a robust approach to quantify the dynamics of epitope presentation^28,29^. However, the high numbers of HLA-restricted peptides available cannot be comprehensively tested to facilitate effective vaccine development. Therefore, we established a rapid and robust screening system combining immune-signature investigation, HLA-peptidomic analysis, and high-throughput immunogenicity testing to evaluate antigen-specific CTL responses (Fig. 1).

## Results

### Relationship between T cell receptor (TCR) diversity and MHC gene expression

**Figure 2:**
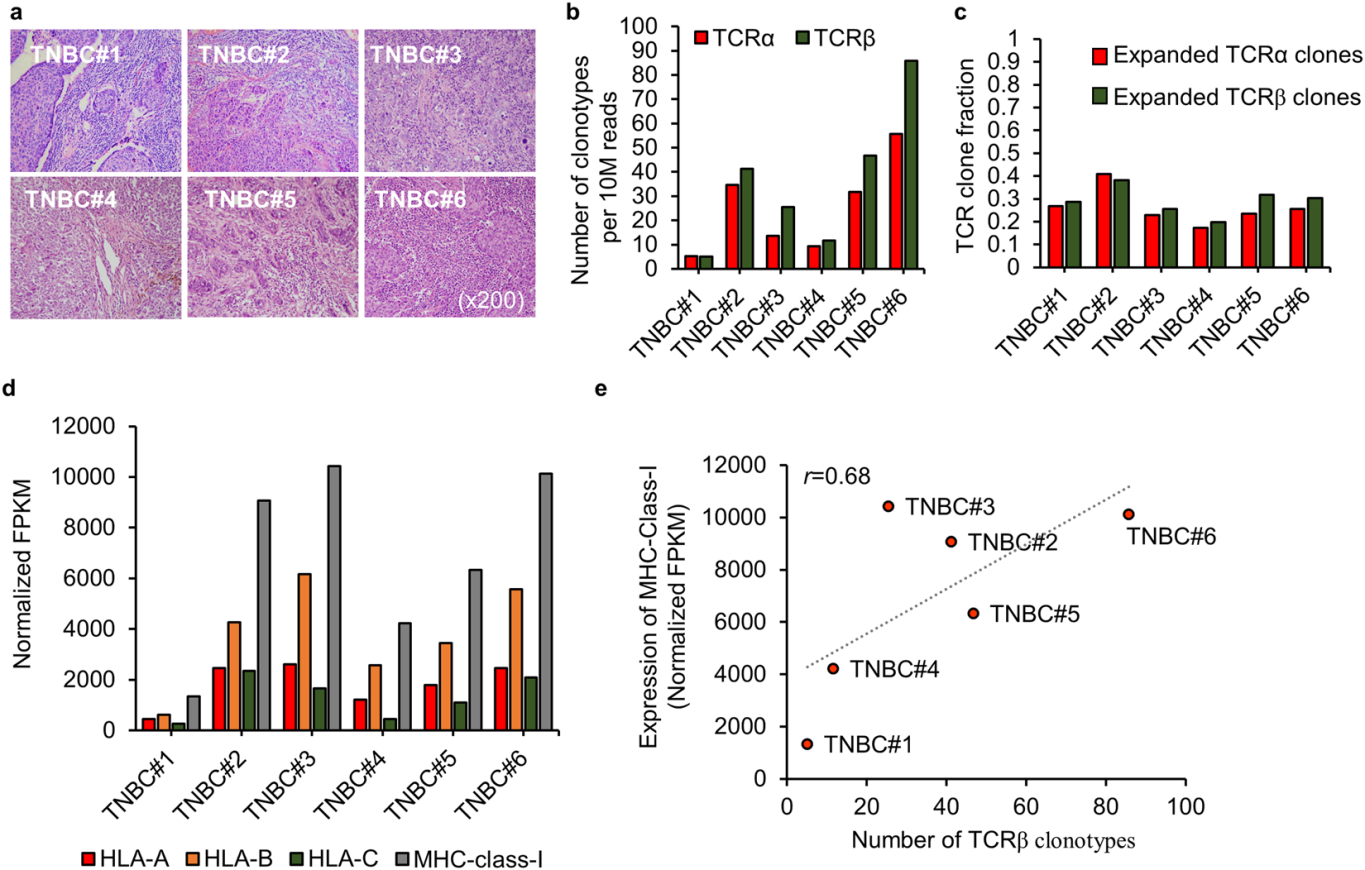
Positive linear correlation between TCR diversity and MHC gene expression. (**a**) Histologic analysis showing TIL-resident tumors from six patients with TNBC. (**b**,**c**) TCR-repertoire analysis showing the number of TCR clonotypes per 10 million reads (**b**) and the clonality of expanded TCR clones defined as the frequency of the top 10% of TCR clones (**c**). (**d**) Comparison of MHC class І expression in tissue samples from each patient. MHC class І expression indicates the total expression of HLA-A, HLA-B, and HLA-C molecules. (**e**) The relationship between the number of TCRβ clonotypes and the expression of MHC class I genes. Pearson’s correlation was calculated between two groups.

TILs (tumor-infiltrating lymphocytes) play a significant role in tumor-sites. Additionally, large amounts of TILs correlate with improved tumor survival^30^; therefore, we preselected TIL-resident TNBC tissues for histologic analysis to identify potentially promising cancer epitopes (Fig. 2a and Supplementary Fig. S1) and scored TIL density by measuring the proportion of the stromal area infiltrated by lymphocytes, as previously described^31^. To select highly immunogenic HLA epitopes, we analyzed the intratumoral heterogeneity of the TCR repertoires in TIL-resident cancers from six patients with TNBC (Fig. 2b,c). The TCR repertoires comprise somatic recombination of the TCRα and β chains, allowing the specificity of each T cell clone to be determined by rearrangement of the V, D, and J segments of the TCRβ chain during generation of the highly variable complementary determining region^32,33^. To evaluate TCR diversity of TILs, we assembled CDR3 sequences using the sequence reads of RNA-seq data. A unique CDR3 sequence of TCRα and TCRβ, respectively, was defined as a clone, and the number of clonotypes represents the number of unique clones per sample after normalization with the corresponding RNA-seq depth (Fig. 2b). The T cell clonal fraction was defined as the frequency of the top 10% of TCRα or TCRβ clones among total TCR clones (Fig. 2c). The top 10 most abundant TCRα and TCRβ sequences in each patient are shown in Supplementary Fig. S2, and expression of the three HLA-class I genes (HLA-A/B/C) is shown in Fig. 2d. We found a linear positive correlation between the number of TCRβ clonotypes and the expression of MHC-class I genes according to Pearson’s correlation coefficient (*r* = 0.68) (Fig. 2e).

### TIL immunoprofiles and immune-specific signatures

**Figure 3:**
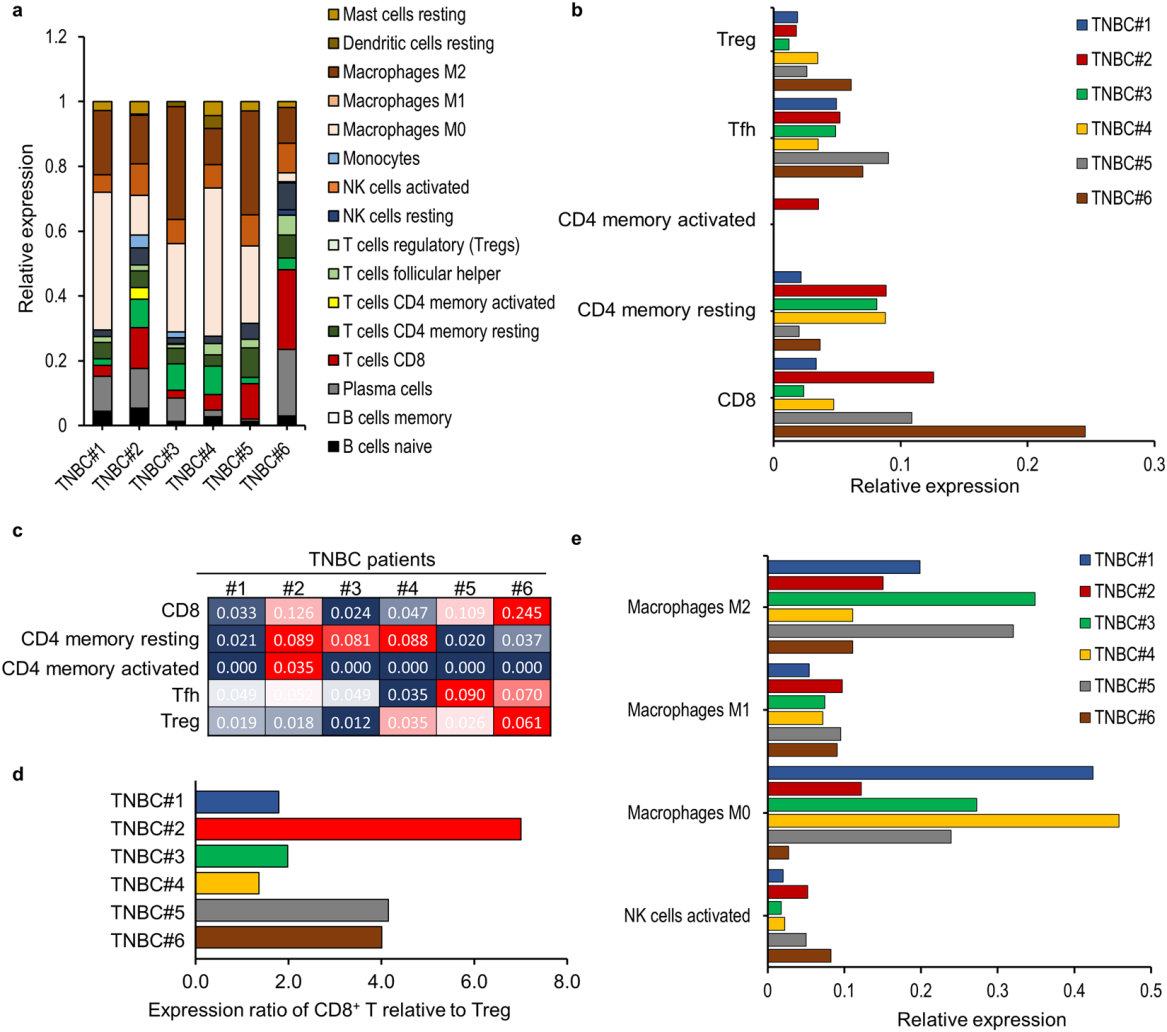
Immune-specific signatures in TILs. Characterization of specific immune-cell types estimated using CIBERSORT. Immune-cell population was evaluated by whole-transcription expression profiles from bulk tissue lysates. (**a**) Overview of immune-cell signatures among TILs. (**b**,**c**) The relative proportion of adoptive T cell-specific signatures among TILs. (**c**) The proportion of CD8^+^ T cells relative to Treg cells. (**d**) The relative proportion of innate immune-cell-specific signatures among TILs.

Several recent studies demonstrated the strong relationship between significant overall survival (OS) and cancer patients harboring a high number of CD8^+^ T cells and a low number of FoxP3^+^ T cells^34^. In particular, the abundance of regulatory T (Treg) cells and macrophages correlated with worse outcomes, whereas the abundance of intratumoral CD8^+^ T cells and CD4^+^ T-helper (Th)1 cells correlated with better prognosis^35^. Additionally, immune-specific signatures in TILs are of potential clinical significance; therefore, we estimated the distribution of TIL types in each patient according to WTS data using CIBERSORT computational analysis^36^ (Fig. 3 and Supplementary Fig. S3). The relative proportion of each infiltrated immune cell was evaluated in each patient by quantifying immune composition from bulk-tissue gene-expression profiles, as enrichment of CD8/CD45RO and Th1 cells are considered positive prognostic factors^37^. The results indicated that CD8^+^ T cells were highly infiltrated in both patients TNBC#2 and TNBC#6, whereas a large proportion of Treg cells was observed in patients TNBC#5 and TNBC#6 (Fig. 3a–c). Patient TNBC#6 displayed a highly enriched frequency of CD8^+^ T cells, as well as Treg cells, suggesting increased accumulation of TILs. Interestingly, we found a significantly increased proportion of CD8^+^ T cells relative to Treg cells in patient TNBC#2 as compared with that observed in other patients (Fig. 3d), suggesting the emergence of promising tumor-associated antigens. On the other hand, we found elevated levels of immune-suppressive macrophages in patients TNBC#1, TNBC#3, and TNBC#5, which is predictive of a negative outcome (Fig. 3e).

### HLA-peptidome and LC-MS/MS results

**Figure 4:**
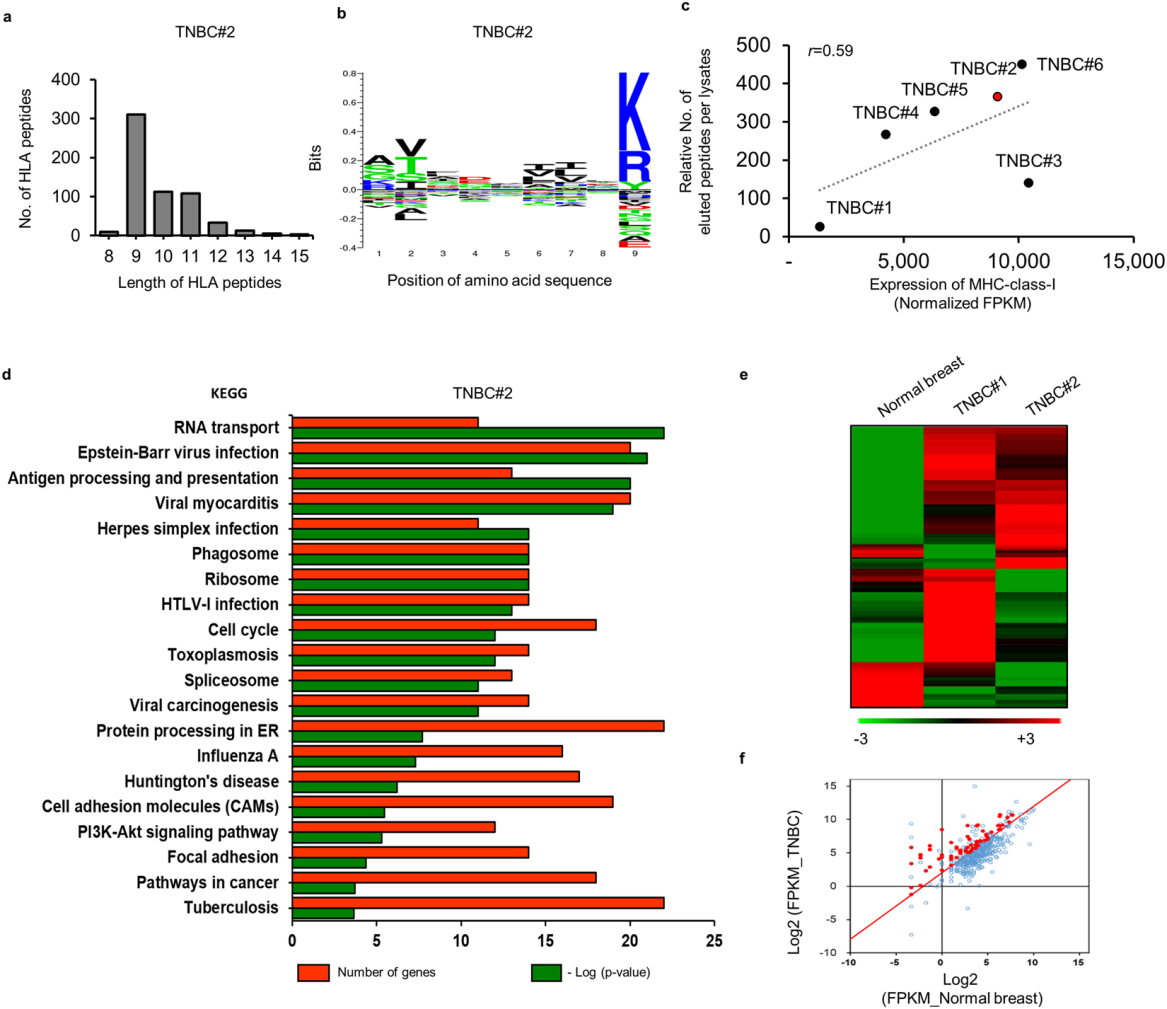
Identification of TNBC-associated HLA peptides by HLA-peptidomics. (**a**) The number and typical length distribution of HLA peptides. (**b**) Logo plot showing sequence analysis of all 9-mer HLA peptides. (**c**) The relationship between the relative number of eluted peptides per lysate and MHC gene expression. Pearson’s correlation was calculated between two groups. (**d**) KEGG enrichment pathway analysis was performed to determine the function of genes derived from correlated HLA peptides. (**e**,**f**) Comparison of the transcription profiles of correlated genes in normal and TNBC tissue in order to remove genes exhibiting low expression (≤2 log_2_ fold change). (**e**) Heatmap created by MultiExperiment Viewer software. (**f**) Red dots in the scatterplot indicate genes exhibiting a fold change of ≥4 log_2_ according to mRNA levels relative to levels observed in normal breast tissue.

To identify naturally existing MHC class I -restricted ligands, we used an immunoproteomic approach involving tissue from six patients with TNBCs and an MHC-І antibody specific for the HLA-A, B, and C molecules (Fig. 4 and Supplementary Fig. S4). After immunoprecipitation, high-resolution LC-MS/MS analysis identified and quantified the HLA peptide sequences with a 1% false discovery rate (FDR) (Supplementary Fig. S5). Notably, the number of eluted peptides from each of the six patients were substantially different (Supplementary Fig. S6), although >96% of the HLA peptides analyzed by LC-MS/MS showed typical properties associated with epitope length. As expected, most of the peptides were nine amino acids long, with only a few having 13 to 15 amino acids, suggesting a high level of consistency (Fig. 4a and Supplementary Fig. S6). Clustering of the 9-mer HLA peptides showed predominant enrichment of residues at peptide positions 2 and 9 and consistent with the anchor motifs required by the binding groove of each HLA molecule^27^ (Fig. 4b and Supplementary Fig. S7). Additionally, we found high numbers of CD8^+^ T and CD4^+^ Th1 cells infiltrating into the tumor sites of patient TNBC#2 and relative to Treg cells, with patient TNBC#2 showing a 4-fold higher number of CD8^+^ T cells as compared with that in patient TNBC#1 and accompanied by the lowest expression of MHC class I genes, suggesting a higher accumulation of antigen-specific CTLs in patient TNBC#2 (Supplementary Figs. S3,S8). A total of 594 peptides were identified from the tissue of patient TNBC#2 along with elevated expression of HLA genes, whereas only five peptides were received from tissue from patient TNBC#1 and all showing the lowest expression of MHC class I genes (Fig. 4c and Supplementary Fig. S8). Moreover, we observed a positive correlation between the number of eluted peptides relative to input lysate and the expression of MHC class I genes (Fig. 4c).

We then performed Kyoto Encyclopedia of Genes and Genomes (KEGG) pathway enrichment analysis to investigate the genes associated with the 594 HLA-binding peptides in patient TNBC#2 and the homotypic HLA-A*11:01 allele (Fig. 4d). Interestingly, the high-count genes (N > 10) were significantly enriched in KEGG pathways related to cancer, protein processing in the endoplasmic reticulum (ER), viral carcinogenesis, and antigen processing and presentation. Numerous cancer-related genes overexpressed in cancer tissues contribute to cancer-specific or associated epitopes^38^, and HLA epitopes require proteasomal digestion and translocation into the ER to bind MHC class-І molecules^39^. It would be expected that the expression of genes encoding machinery responsible for antigen processing would be elevated under these circumstances. These results suggested that the eluted HLA peptides identified were naturally presented by HLA molecules. We further investigated the levels of the eluted peptides based on RNA-seq analysis of corresponding mRNA from the same sample (Fig. 4e,f). Compared with normal breast tissues, 174 of 594 peptides corresponding to proteins from the same sample showed elevated abundances in cancer tissues accompanied by significant differences in mRNA expression (≥2 log_2_ fold change). Subsequent *in silico* prediction of the HLA-binding affinities to the 174 HLA peptides and calculation of their respective binding affinity to specific alleles (predicted IC_50_)^40^ yielded a list of the top 20 highest ranking peptides derived from patient TNBC#2 (Table 1).

**Table 1:** Top 20 immunogenic CTL peptide candidates with scores were generated from a prediction tool, NetMHC.

Rank-ing	Gene	Eluted Peptide	IC50 (nM)	Immuno-genicity score	HLA type	Rank-ing	Gene	Eluted Peptide	IC50 (nM)	Immuno-genicity score	HLA type
1	DYNLRB1	SLMHSFILK	5	0.030	A11:01	11	LDHA	GSLFLRTPK	12	0.178	A11:01
2	PRKDC	STFDTQITK	6	0.124	A11:01	12	PRKDC	STFDTQITKK	12	0.017	A11:01
3	NDUFC2	KTYGEIFEK	8	0.413	A11:01	13	PSMD14	AAMLDTVVFK	12	0.133	A11:01
4	EIF4A1	GIYAYGFEK	8	0.224	A11:01	14	LDHB	GSLFLQTPK	14	0.021	A11:01
5	PSMD14	AMLDTVVFK	8	0.199	A11:01	15	AURKB	KSHFIVALK	14	0.323	A11:01
6	DHCR7	AVSTFAMVK	9	0.012	A11:01	16	BCAP31	GAMEHFHMK	14	0.110	A11:01
7	TOMM5	RVTPFILKK	9	0.105	A11:01	17	RPL24	ASLADIMAK	15	0.057	A11:01
8	ROPN1B	SALGVTITK	10	0.242	A11:01	18	EIF3H	STYYGSFVTR	19	0.035	A11:01
9	TUBB	STAIQELFK	11	0.187	A11:01	19	NDUFA11	GTFLEGVAK	19	0.214	A11:01
10	TCP1	GVFEPTIVK	11	0.301	A11:01	20	C3	ATFGTQVVEK	19	0.129	A11:01

### A rapid imaging-based screening method to determine antigen-specific T cell response at the single-cell level

**Figure 5:**
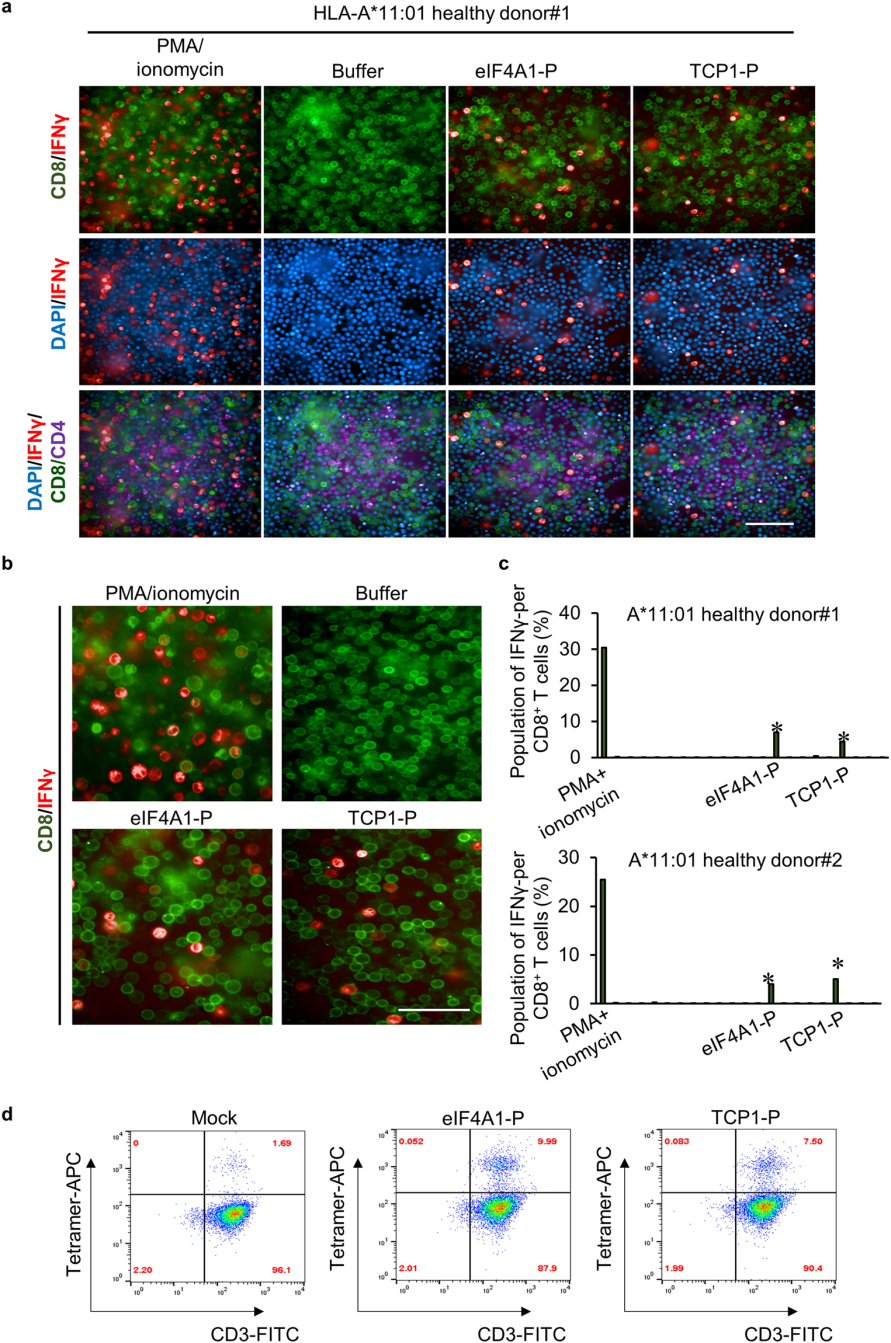
Identification of IFNγ response against peptide-reactive T cells using high-throughput imaging. (**a**) The 384-well screening system used to detect IFNγ-producing CD8^+^ T cells. (**b**) Detection of intracellular IFNγ levels in CD8^+^ T cells following peptide administration. PMA and ionomycin were used for non-specific T cell stimulation. Representative images are shown. (**c**) Bar graphs depicting the frequency of antigen-reactive CD8^+^ T cells. *P < 0.005, one-way analysis of variance, followed by Tukey’s multiple comparison test. (**d**) Peptide/A*11:01 MHC-tetramer staining for antigen-specific T cells analyzed by flow cytometry.

To determine whether the experimentally identified peptides can functionally elicit an immune response, we evaluated cytokine production by the CD8^+^ T cells. Currently, intracellular cytokine staining (ICS)-based detection methods for monitoring *ex vivo* IFNγ response show low throughput relative to the number of candidate antigens being tested. Moreover, an individual antigen test requires large amounts of immune cells^41^. Therefore, we developed an efficient and comprehensive screening system to test CTL response based on a high-content, high-throughput imaging approach (Supplementary Fig. S9). This fluorescence-imaging-based screening system allows the use of lower numbers of viable cells up-scaled performance^42,43^. Development of a 384-well format capable of screening mixed populations of T cells for their response against large number of peptides enables a cost-effective approach to phenotype analysis. Notably, cancer-associated antigens are highly attractive targets for determining their efficacy in triggering a T cell response; however, numerous clinical trials targeting TAAs for vaccine development have failed to demonstrate clinical efficacy due to immune self-tolerance. To identify highly immunogenic peptides incapable of eliciting self-tolerance, we tested the antigen-specific T cell response in PBMCs from a healthy donor. Monitoring *ex vivo* IFNγ-producing PBMC reactivity using our fluorescence-labelled cell-based screening system (Fig. 5a–c, Supplementary Figs. S10,S11) revealed significant IFNγ responses to two individual epitopes (eIF4A1-P and TCP1-P) in CD8^+^ T cells labelled with a FITC conjugated anti-human CD8 antibody (Fig. 5b). Additionally, treatment with the HLA-A*11:01-specific epitopes allowed detection of APC-conjugated IFNγ released from CD8^+^ T cells (Fig. 5a,b), with PMA and ionomycin co-treatment used to trigger T cell activation as a positive control. Similarly, PBMCs from two of the three healthy donors were reactive against same two epitopes (Fig. 5c).

To further analyze the peptide-induced CD8^+^ T cells, we generated HLA-A*11:01 tetramers targeting specific peptide-reactive CD8^+^ T cells. FACS analysis revealed that 9.99% and 7.50% of T cells were targeted by eIF4A1-P and TCP1-P, respectively, and detectable on day 12 of *ex vivo* T cell expansion (Fig. 5d). These findings suggested the efficacy of our method to screen highly immunogenic CTL epitopes using an imaging system on the detection of intracellular IFNγ levels following peptide stimulation. The two genes associated with the peptide epitopes, the translation initiation factor *eIF4A1* and *TCP1*, a member of the chaperonin-containing complex TCP1-containing ring complex (TRiC), are involved in tumor proliferation and survival. eIF4A1 controls translation initiation and is a critical checkpoint protein involved in cell proliferation and tumorigenesis^44^. Additionally, TCP1, as a TRiC member, is involved in tumor survival and growth and an oncogene driver^45^. Moreover, these two genes are significantly overexpressed in tumor tissues according to WTS data and data from the Genotype-Tissue Expression Project public proteomic database, suggesting their potential as therapeutic targets. These results identified two epitopes derived from eIF4A1 and TCP1 as potential promising immunogenic antigens to boost T cell response.

## Discussion

Patients with TNBC, which is defined by the absence of estrogen receptor, progesterone receptor, and human epidermal growth factor receptor 2, have a higher tendency for recurrence at ~3 years after diagnosis^46,47^. There are currently no known therapeutic targets for TBNC patients due to the molecular heterogeneity of the disease^48^. Recent accumulation of massive and comprehensive bioinformatics data allowed identification of potential therapeutic targets associated with clinical survival^49^. Interestingly, a TNBC subtype characterized by high levels of immune genes involved in T cell function, immune response, and antigen processing, was found to be associated with favorable prognosis, suggesting a close correlation between immune-gene signatures and better clinical outcomes^50^. Therefore, it is possible that TILs controlling clinical cancer progression represent key factors for preselection of tumors prior to HLA-immunopeptidomics. In this study, TIL-resident tissues were pre-selected comprehensively to investigate a diversity of TCR repertoires and immune profile as predictors of clinical outcome. We then found a positive correlation between TCR diversity, reflecting clonal composition, and the expression of MHC- class І molecules, suggesting that active tumor-antigen presentation promotes the generation of antigen-specific TILs. Additionally, immune-specific signature analysis can discriminate specific immune-cell types in each patient and thus enhance the efficiency of selective HLA-peptidomic approaches. Notably, the expressional comparison of CD8^+^ T cells relative to immune-suppressive Treg cells is extremely crucial to select the high antigenicity of antigens reflecting the therapeutic efficacy. Thus, the extreme increase in the number of CD8^+^ T cells relative to that of Treg cells in patient TNBC#2 presented a major analytical reason for further *in vitro* T cell response testing. Furthermore, our results indicated a positive correlation between the number of peptides identified via HLA-peptidomics and the amount of HLA molecules expressed on the surface of cancer cells. It is also possible that the observed difference in the number of eluted peptides might have been influenced by HLA-expression levels resulting from active induction of antigen presentation on MHC molecules, which subsequently elicited a strong immune response. Thus, these findings suggested that several factors should be considered for successful HLA-peptidomic approaches influenced by TCR diversity and elevated expression of HLA genes.

Additionally, we showed that integrating HLA-peptidomics with imaging-based immunogenicity screening is applicable for the discovery of highly immunogenic CTL epitopes. Characterization of antigen-specific cellular immune response is essential to confirm vaccine-related effects specific to a cancer antigen. Currently, there are only few assays (e.g., the enzyme-linked immunosorbent spot assay) capable of quantifying T cell responses^51,52^, and the choice of which assay to use depends on the experimental scale, cost, equipment, reproducibility, and required detection sensitivity. A high-throughput imaging system provides an optimal platform for highly sensitive and quantitative analysis of individual T cells at the single-cell level. Moreover, ICS-based cytokine detection allows identification of specific cell subpopulations, even when using a small number of cells.

The immunopeptidome approach serves massive HLA-associated peptides as the collection of cancer epitopes; however, there are obstacles to rapidly determining the optimal set of promising epitopes by testing an enormous pool of peptide candidates in a single measurement. In this study, the HLA-peptidomics approach combined with comprehensive analysis of immune-specific signatures and TCR repertories showed high selectivity to determine the immunogenic T-cell epitopes. Sequentially, the high-content imaging system allowed high-resolution analysis for T cell reactivity.

Despite the need for discovery of tumor-derived antigens for effective cancer vaccine development, selection of antigens that elicit robust immune response remains challenging. Here, we report a smart strategy for streamlined selection of cancer antigens in vaccine development. Through integrative multi-omics and high-content cell imaging, we identified highly immunogenic epitopes from patients with TNBC. Identification of potential vaccine epitopes coupled with immune-specific signature analysis, HLA-peptidomics, and single-cell-based immunogenicity testing offers a discriminative and powerful tool for cancer-vaccine development.

## Methods

### Patients and ethics statement

Tumor samples were collected from six different patients with TNBC who were diagnosed at stage I/II at the Korea University Anam Hospital (Seoul, Korea) and Asan Medical Center (Seoul, Korea). For patients TNBC#1 and TNBC#2, the biospecimens and data used in this study were provided by the Biobank of Korea University Anam Hospital (BBB2017-03-01). The ethics approval of this study was granted by the IRB committee at the Korea University Anam Hospital (BBB2017-03-01). Patients provided written informed consent for the collection and analysis of their tumor samples, and this project was reviewed and approved by the Institutional Review Board of the Korea University Anam Hospital (2017AN0399). Hematoxylin and eosin-stained samples were reviewed and selected by a breast pathologist based on the tumor-infiltrating lymphocyte (TIL) score as the proportion of the stromal area infiltrated by lymphocytes and according to the recommendations of the International TIL Breast Cancer Working Group^31^. Four patients-derived tissues (TNBC#3–#6) and peripheral blood mononuclear cells (PBMCs) from three healthy donors were obtained from Asan Medical Center, with requirements for informed donor consent for the use of data followed and approved by the Institutional Review Board (2017-0784 and 2016-0935) of Asan Medical Center. Healthy Donors also provided written informed consent for the collection and analysis of their PMBCS. All methods were carried out in accordance with relevant guidelines and regulations, approved by the institutional review board of Korea University Anam Hospital and Asan Medical center.

### Analysis of high-throughput sequencing

DNA and RNA were simultaneously extracted from cryo-pulverized TNBC tissue powder using an AllPrep kit (Qiagen, Hilden, Germany), and libraries for whole-exome sequencing and total RNA sequencing, respectively, were prepared using TruSeq library prep kits (Illumina, San Diego, CA, USA). The libraries were sequenced using the HiSeq platform (Illumina), and raw data were mapped onto hg38 using the bwa mem algorithm (http://bio-bwa.sourceforge.net/). Variant calling was performed using the Genome Analysis Toolkit (https://software.broadinstitute.org/gatk/) as previously described^53^. A custom proteogenomic search database was generated for the variants using the proteomics tool QUILTS (http://www.fenyolab.org/tools/tools.html). For WTS data, contaminating adapters and low-quality bases were removed with Trimmomatic^54^, and the trimmed data were mapped onto hg38 using STAR (version 2.5.3a)^55^. Gene expression, including that of HLA genes, was calculated by RSEM (version 1.3.0)^56^, and HLA presentation on multiple cancer cells was evaluated using the Expression Atlas (https://www.proteinatlas.org/humanproteome/tissue/cancer)^57^. For identification and quantification of TILs from WTS data, we used MiXCR (v.2.1.3; https://mixcr.readthedocs.io/en/master/) with the alignment parameter *-p rna- seq*.^58^. HLA typing at 4-digit resolution was performed using the HLAscan method and WES data (Synthekabio, Korea).

### Purification of HLA-class I peptides

The LC-MS/MS analysis was performed as previously described^59^. HLA-class I peptides were purified from TNBC tissues, and frozen tissues were pulverized with a CP02 cryoPREP automated dry pulverizer (Covaris, Woburn, MA, USA), followed by incubation at 4 °C for 1 h with lysis buffer containing 0.25% sodium deoxycholate, 0.2 mM iodoacetamide, 1 mM EDTA, 1 mM PMSF, 1% octyl-β-D-glucopyranoside (Sigma-Aldrich, St. Louis, MO, USA), and a protease-inhibitor cocktail (Roche, Mannheim, Germany) in phosphate-buffered saline (PBS). The lysates were cleared by centrifugation for 20 min at 15,000 *g* and 4 °C. HLA-class I molecules were purified using the W6/32 monoclonal antibody bound to Amino-Link beads (Thermo Scientific, Waltham, MA, USA), as previously described^60^. The anti-HLA-class I antibody was purchased from Abcam (Cambridge, UK). HLA-peptide complexes were eluted from the affinity column using five column volumes of 0.1 N acetic acid. The eluted HLA-class I proteins and the released peptides were loaded on Sep-Pak tC18 columns (Waters, Milford, MA, USA), and the peptide fraction was eluted with 30% acetonitrile in 0.1% trifluoroacetic acid, followed by drying by vacuum centrifugation.

### LC-MS/MS analysis of HLA-class I peptides

The LC-MS/MS analysis was performed as previously described^59^. Dried peptide samples were reconstituted in 10 µL of 0.1% formic acid, and an aliquot containing ~4 μL was injected from a cooled (10 °C) autosampler into a reversed-phase Magic C18aq column (15 cm × 75 μm (packed in-house); Michrom BioResources, Auburn, CA, USA) on an Eksigent nanoLC 2D system at a flow rate of 300 nL/min. Prior to use, the column was equilibrated with 95% buffer A (0.1% formic acid in water) and 5% buffer B (0.1% formic acid in acetonitrile). The peptides were eluted with a linear gradient from 5% to 30% buffer B over 70 min and 30% to 70% buffer B over 5 min, followed by an organic wash and aqueous re-equilibration at a flow rate of 300 nL/min, with a total run time of 95 min. The high-performance liquid chromatography system was coupled to an LTQ-Orbitrap XL mass spectrometer (Thermo Fisher Scientific, Bremen, Germany) operated in data-dependent acquisition mode. Full scans (m/z 300–1800) were acquired at a resolution of 60,000 using an automatic gain-control (AGC) target value of 1e6 and a maximum ion-injection time of 10 ms. Tandem mass spectra were generated for up to 5 precursors by collision-induced dissociation in the ion-trap using a normalized collision energy of 35%. The dynamic exclusion was set to 60 s, and fragment ions were detected at a normal scan mode using an AGC target value of 1e5 and a maximum ion-injection time of 500 ms. Source ionization parameters were as follows: spray voltage, 1.9 kV; and capillary temperature, 275 °C.

### Data analysis of the HLA-class I peptidome

MS data were analyzed using MaxQuant software (v.1.5.8.3)^61^ against the UniProt database (April 40, 2016; https://www.uniprot.org/), and personalized human database from patient-derived WES data. N-terminal acetylation and methionine oxidation were set as variable modifications, enzyme specificity was set as “unspecific,” and FDRs for peptides and proteins were set at 0.01 and 1, respectively. Possible sequence matches were restricted to eight to 15 amino acids, a maximum peptide mass of 2000 Da, and a maximum charge state of three. Main search peptide tolerance was set at 10, and the box for “Use MS2 centroids” was checked. Hits to the reverse database and contaminants were removed from the “peptide.txt” output file produced by MaxQuant. Peptides were subjected to HLA-class I binding and immunogenicity prediction analyses using NetMHC (http://www.cbs.dtu.dk/services/NetMHC/) and the MHC I immunogenicity portion of the IEDB Analysis Resource (http://tools.iedb.org/immunogenicity/), respectively. The logo-plots were constructed using a Seq.2Logo method^62^.

### Rapid single cell-based imaging analysis of T cell reactivity

The IFNγ response was evaluated in PBMCs isolated from three HLA-A*11:01-positive healthy donors. T cell responses to treatment with individual peptides were monitored after 12 days of *in vitro* culture, as previously described^11^. Briefly, PBMCs were cultured in RPMI-1640 supplemented with L-glutamine, non-essential amino acids, HEPES, β-mercaptoethanol, sodium pyruvate, penicillin/streptomycin (Gibco; Thermo Fisher Scientific), and 10% human AB serum (Gibco), with a total of 5 × 10^4^ PBMCs used in each well. For antigen-specific T cell expansion, individual peptides (2 µg/mL) were incubated with PBMCs in the presence of interleukin (IL)-7 (20 ng/mL; Peprotech, Rocky Hill, NJ, USA) for 3 days. Each peptide with >95% purity was synthesized by Synpeptide (Shanghai, China). For non-specific T cell expansion, T cells were stimulated using a T cell activation/expansion kit (Miltenyi Biotec, Bergisch Gladbach, Germany), and cells were cultured every 3 days by replacing the medium with fresh half-medium containing IL-7 (5 ng/mL) and IL-15 (5 ng/mL; Peprotech). On day 12, for *ex vivo* intracellular IFNγ detection, each peptide (10 µg/mL) or phytohemagglutinin (PMA) (50 ng/mL; Sigma-Aldrich) plus ionomycin (1 µg/mL; Sigma-Aldrich) for the positive control was administered in the presence of Brefeldin A (1:1000; BioLegend, San Diego, CA, USA) for overnight incubation according to the ICS protocol. After intracellular fixation using BD Cytofix/Cytoperm fixation/permeabilization kit (BD Biosciences, San Jose, CA, USA), cells were stained with antibodies against surface markers and IFNγ at 4 °C for either 1 h or overnight. For visualization of IFNγ-producing T cells, a fluorescein isothiocyanate (FITC)-conjugated anti-human CD8α antibody (R&D Systems, Minneapolis, MN, USA), an Alexa594-conjuated anti-human CD4 antibody (BioLegend), and an allophycocyanin (APC)-conjugated anti-human IFNγ antibody (BioLegend) were specifically labeled for CD8^+^ T, CD4^+^ T, and intracellular IFNγ capture, respectively. Cells were then washed with PBS buffer, and high-throughput imaging was performed using the Operetta CLS high-content analysis system equipped with Harmony software (PerkinElmer, Waltham, MA, USA).

### Detection of antigen-specific T cells

To generate the p/MHC tetramer, a biotinylated HLA-A*11:01 monomer complexed with each peptide was obtained from ImmunoMAX Co., Ltd. (Seoul, Korea). Peptides with >95% purity were synthesized by Synpeptide (China), and their sequences are provided in Table 1. To generate an APC-labeled p/MHC complex tetramer, p/MHC complex monomers were tetramerized in the presence of APC- conjugated streptavidin (BD Biosciences). T cells were then stained with 1 ng/µL of the p/MHC tetramer in Fluorescence-activated cell sorting (FACS) buffer (BioLegend) for 10 min at room temperature, and after washing, immunofluorescence was detected by FACS. T cells were gated according to the populations of cells labeled with the FITC-conjugated anti-human CD3 antibody (BioLegend).

## Supplementary information

Supplementary Information.
