# Supplementary material for: Streamlined selection of cancer antigens for vaccine development through integrative multi-omics and high-content cell imaging
Source: Sci Rep. 2020 Apr 3;10:5885. doi: 10.1038/s41598-020-62244-z (PMC7125174; doi:10.1038/s41598-020-62244-z)
Supplement: Supplementary file 1 — Supplementary Information. [file 41598_2020_62244_MOESM1_ESM.pdf]

Supplementary Information

Streamlined selection of cancer antigens for vaccine development through integrative multi-omics and high-content cell imaging

Ki-Cheol Han, Daechan Park, Shinyeong Ju, Anna Ju, Sun-Hee Heo, Young-Ae Kim, Young Eun Lee, Ji Eun Lee, Kyong Hwa Park, Se-Ho Park, Hee Jin Lee, Cheolju Lee and Mihue Jang

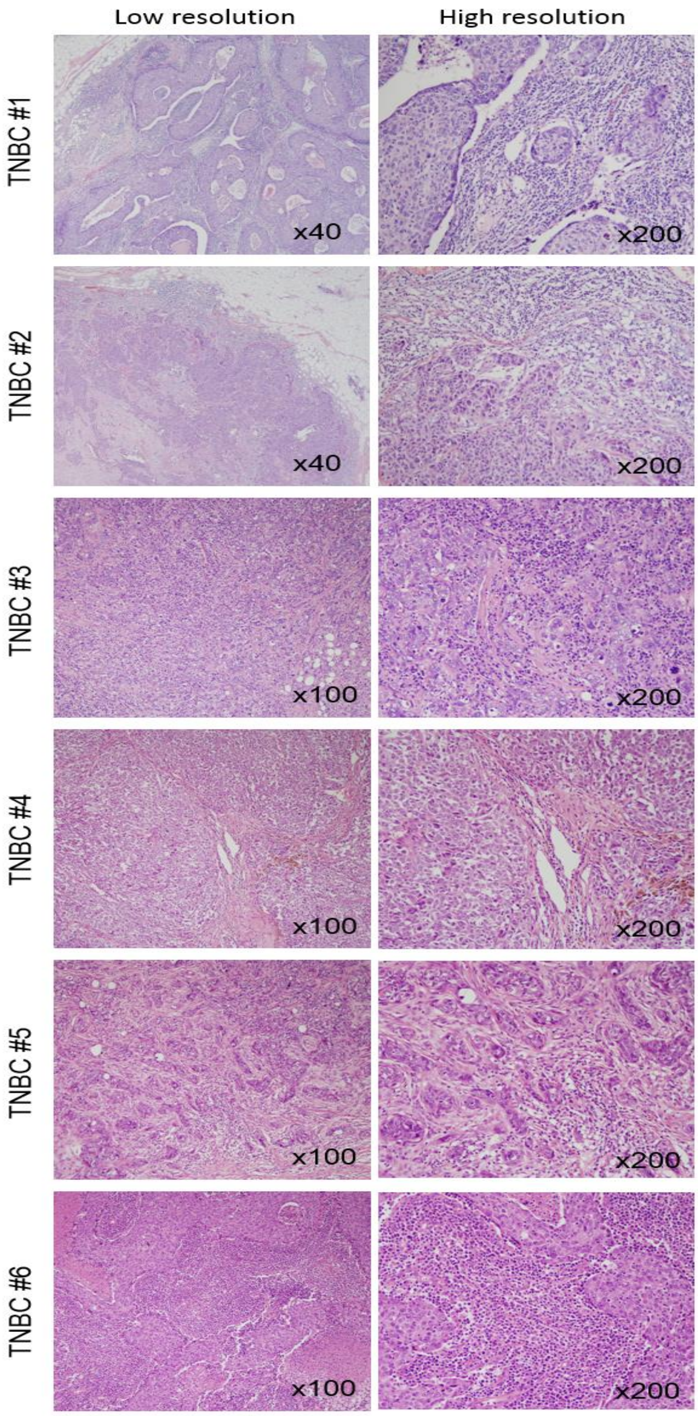

Figure S1. TIL identification in TNBC tissues. Hematoxylin and eosin staining showing the presence of TILs in TNBC tissues. The proportion of the stromal area infiltrated by lymphocytes was measured to obtain a TIL-density score.

TNBC#1			
TCRα		TCRβ	
cloneFraction	aaSeqCDR3	cloneFraction	aaSeqCDR3
0.0732	CAGTLINQAGTALIF	0.0957	CASSLGSKRSSYNEQFF
0.0610	CIVIRTGSARQLTF	0.0426	CASSTRDRHFYNEQFF
0.0366	CALDPSISATNKLIF	0.0426	CASSERASGRDNEQFF
0.0366	CLVGDRKNQGGKLIF	0.0426	CASSLSGLPGYTF
0.0366	CAVGGPGNFNKFYF	0.0319	CASSPADNTDTQYF
0.0244	CAVSLGDTSGTDKLIF	0.0319	CSASGVRPDTQYF
0.0244	CVVSRFSGNTPLVF	0.0319	CSAYKSQETQYF
0.0244	CAVRSSGSARQLTF	0.0213	CASSLAPTGGPYEQYF
0.0244	CVVSSYNTDKLIF	0.0213	CASSLYLSGANVLTF
0.0244	CAASKGGYQKVTF	0.0213	CASSPRPFSNQPQHF

TNBC#2			
TCRα		TCRβ	
cloneFraction	aaSeqCDR3	cloneFraction	aaSeqCDR3
0.1185	CLVAAGYSTLTF	0.0390	CASSQTLDREEGTDTQYF
0.0488	CAFANNNDMRF	0.0390	CSVPNLAGGSYNEQFF
0.0223	CAVKTSYDKVIF	0.0264	CASSLLAGGHNEQFF
0.0153	CATDDELGKLVF	0.0229	CASRGGSPYEQYF
0.0139	CAVSDYNQGGKLIF	0.0218	CASSYSTVYGYTF
0.0112	CAVVELAGNNRKLIW	0.0115	CASSLERGTEAFF
0.0098	CLVAHRGSSNTGKLIF	0.0103	CASSSRTVYNEQFF
0.0084	CAVTRGR_FGNVLHC	0.0092	CAISETWTSGRQTQYF
0.0084	CAVNEGGGSYIPTF	0.0092	CASSTPGTGAGELFF
0.0084	CAPGGKRALTF	0.0092	CASSQVTNTEAFF

TNBC#3			
TCRα		TCRβ	
cloneFraction	aaSeqCDR3	cloneFraction	aaSeqCDR3
0.0550	CAMSGGSGAGSYQLTF	0.0335	CASSETTGALQFF
0.0367	CAVQRQGGSEKLVF	0.0209	CASSLRRGLPPETQYF
0.0275	CALSFPSGGSYIPTF	0.0209	CASSRLGSSYNEQFF
0.0275	CAERMDSSYKLIF	0.0209	CASSTTAGNTIYF
0.0275	CAFNNAGNMLTF	0.0167	CASTVAGTGLRNEQFF
0.0183	CAAIRIEAVYSGGGADGLTF	0.0167	CASSLDLNRENTEAFF
0.0183	CVVSGRSGAGSYQLTF	0.0167	CSARDRAGGSRETQYF
0.0183	CAYRGITAGTASKLTF	0.0167	CASRPPTGRHSPLHF
0.0183	CALDPFTGGGNKLTF	0.0167	CASKASGWEDTQYF
0.0183	CAVAQRGGATNKLIF	0.0126	CASSPRGGLAGGLNTGELFF

TNBC#4			
TCRα		TCRβ	
cloneFraction	aaSeqCDR3	cloneFraction	aaSeqCDR3
0.0435	CATDSLVAGNNRKLIW	0.0492	CASSTPWGTAVSYEQYF
0.0435	CVVTHFGGFKTIF	0.0492	CASSPPVRAYNEQFF
0.0435	CVVSAKSNYQLIW	0.0328	CSANQLSTSGRWYNEQFF
0.0435	CAMSHFGNEKLTF	0.0328	CASSRGGASYNEQFF
0.0217	CALSEARETSYDKVIF	0.0328	CASSPPVREYGYTF
0.0217	CAVRDKGSGNTGKLIF	0.0328	CASSSPGQGVGYTF
0.0217	CALRLDIQGAQKLVF	0.0328	CATNEQGGEQFF
0.0217	CAMREANTNAGKSTF	0.0164	CASSTGGGGPLLALQETQYF
0.0217	CAMREDYNAGNMLTF	0.0164	CATSDLMTSGANTGELFF
0.0217	CAYRGVQGAQKLVF	0.0164	CASSPYLLQDMGYNEQFF

TNBC#5			
TCRα		TCRβ	
cloneFraction	aaSeqCDR3	cloneFraction	aaSeqCDR3
0.0288	CAYRTARGSQGNLIF	0.0619	CASGRSYEQYF
0.0288	CAVDADGGSQGNLIF	0.0229	CASSLQRGNNEQFF
0.0165	CAGQEVGAGSYQLTF	0.0183	CASSQVRGRQETQYF
0.0123	CASPASGGSNYKLTF	0.0183	CASSLEQGFPQFF
0.0123	CAVSDSGGSNYKLTF	0.0183	CAISRGYEQYF
0.0123	CAVSHNNAGNMLTF	0.0138	CASSLGTSGGPGNEQFF
0.0123	CAMNVDTGRRALTF	0.0138	CSGTGLEEQFF
0.0123	CVVNSNNFNKFYF	0.0115	CASSRNRGPYEQYF
0.0123	CATGDTGRRALTF	0.0115	CASSRSYEQYF
0.0123	CAETSQAGTALIF	0.0092	CASSEMPRAGTEYGYTF

TNBC#6			
TCRα		TCRβ	
cloneFraction	aaSeqCDR3	cloneFraction	aaSeqCDR3
0.0256	CAMETNRDDKIIF	0.0189	CASSLTGDSNQPQHF
0.0230	CAYRTGTASKLTF	0.0174	CASSFGSSYEQYF
0.0205	CAVTGNQFYF	0.0174	CASSQSNAYEQYF
0.0128	CAPREGTGRRALTF	0.0174	CSGRGSYNEQFF
0.0102	CAVRGRIGSARQLTF	0.0131	CASSLGGLLYNEQFF
0.0102	CAESVGYGGQNFVF	0.0131	CASSLAGLAINEQYF
0.0102	CAMRSGGSYIPTF	0.0102	CASSLLLGGGNTQYF
0.0077	CVVSALSGGGADGLTF	0.0087	CSASGPGKGDYEQYF
0.0077	CAVSASSGGSYIPTF	0.0087	CASSLTSSYNEQFF
0.0077	CAMDAGGTSYGKLTF	0.0087	CASSRGTPNEQFF

Figure S2. Top 10 most abundant TCRα and TCRβ sequences in each TNBC patient.

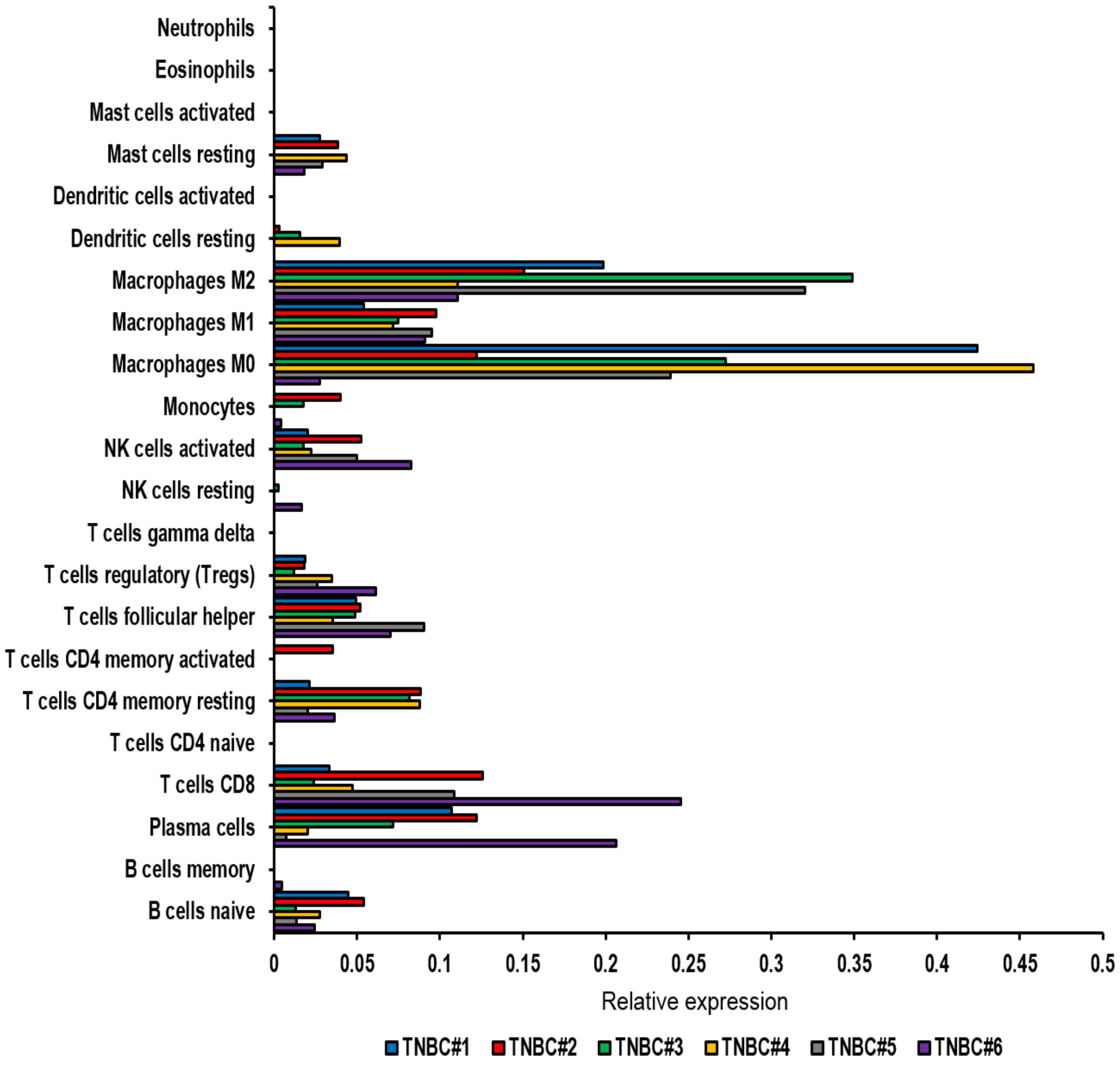

Figure S3. Immune composition of TIL-resident tissue. The immune composition of TIL-resident tissues from each TNBC patient was estimated using CIBERSORT. The relative proportion of specific immune cells was characterized using RNA-seq data.

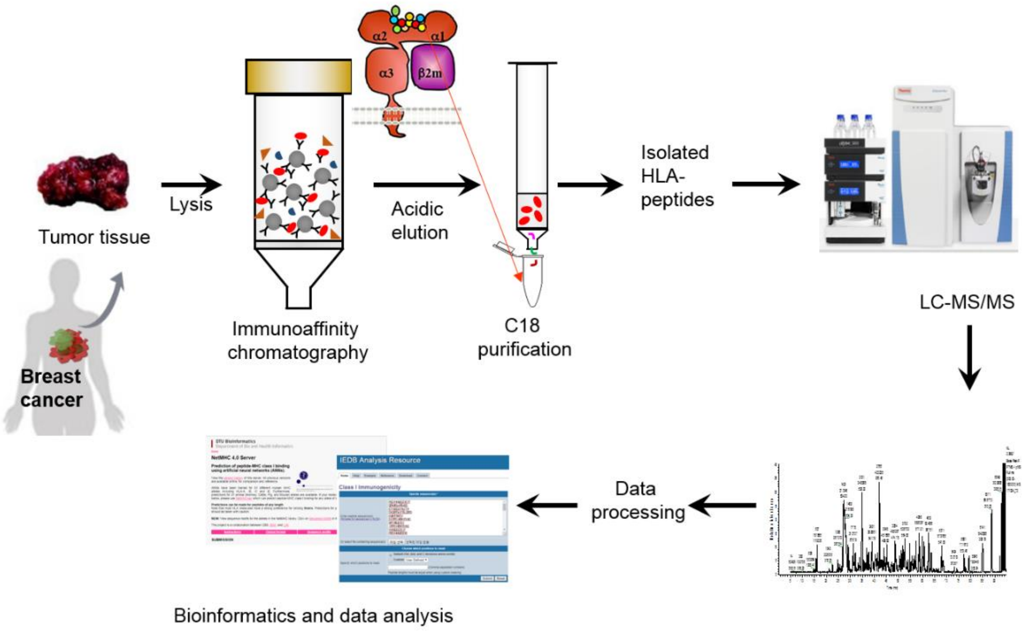

Figure S4. Overview of experimental HLA-peptidomics. HLA class I molecules were purified from lysates of TNBC tissues using the W6/32 monoclonal antibody bound to Amino-Link beads. HLA-peptide complexes were eluted from the affinity column, followed by LC-MS/MS analysis, with the identified peptides subjected to HLA class I binding and immunogenicity prediction using NetMHC and the IEDB Analysis Resource, respectively.

eIF4A-1_GIYAYGFEK (46-54 a.a)

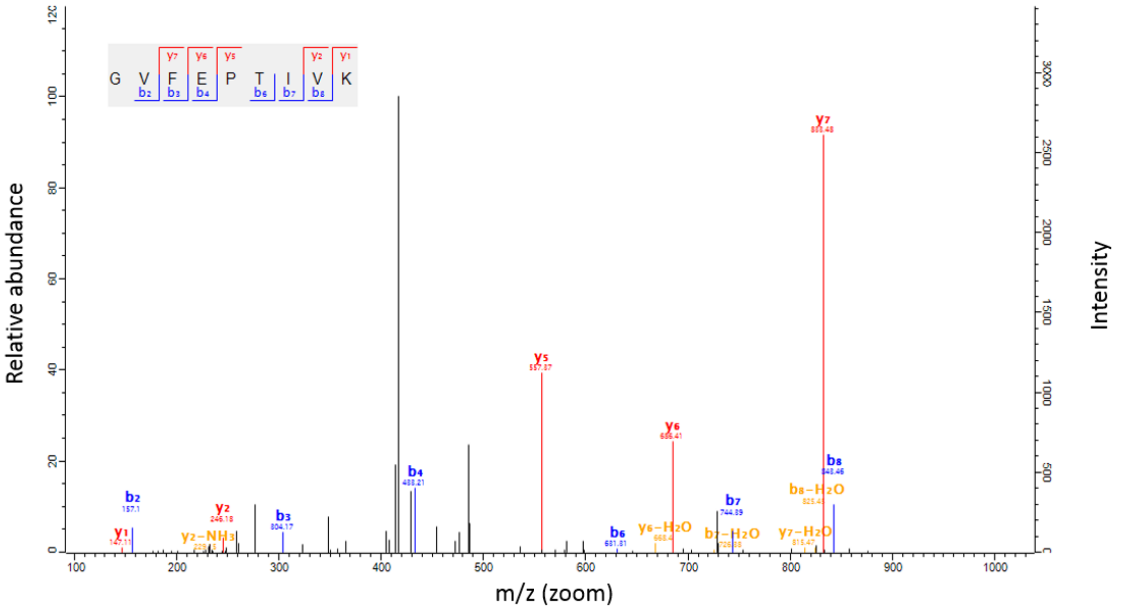

TCP1_GVFEPTIVK (502-510 a.a)

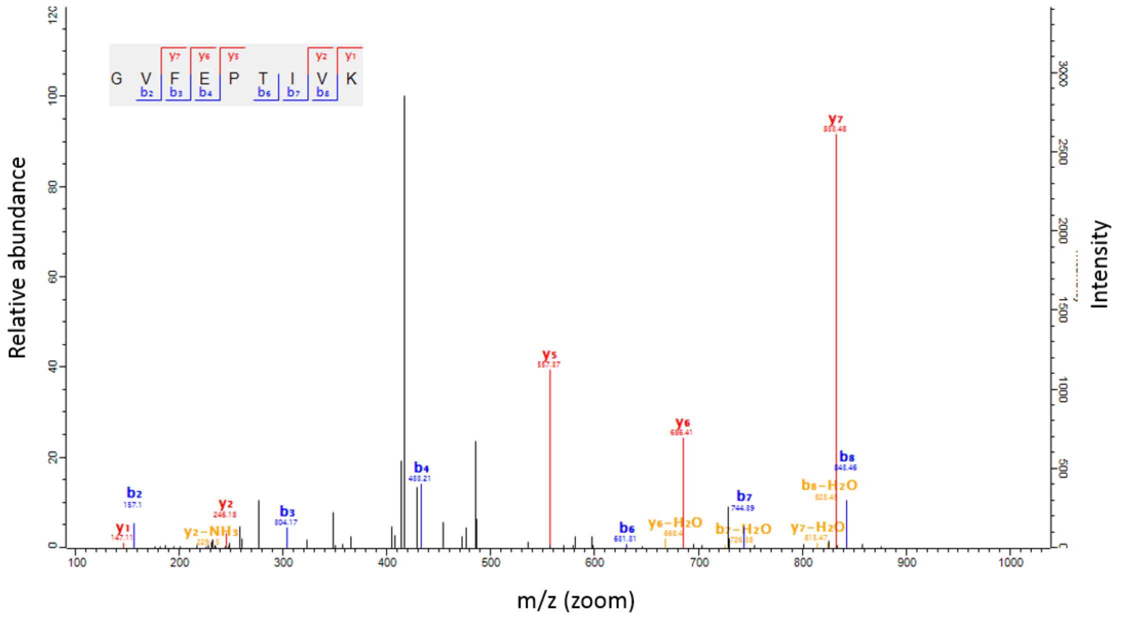

Figure S5. MS/MS spectra of representative eluted peptides from tissue from patient TNBC#2. Peptide locations within the sequences of the matched proteins are marked with curved brackets.

a

Patient ID	TNBC#1 (KUAH2017-03-03)	TNBC#2 (KUAH2017-03-02)	TNBC#3 (AMCT4)	TNBC#4 (AMCT5)	TNBC#5 (AMCT6)	TNBC#6 (AMCT7)
No. peptides (mg of lysates)	5 (29)	594 (41)	743 (353)	1686 (261)	1444 (408)	1749 (182)
HLA types	A*02:06 A*03:01	A*11:01 A*11:01	A*02:06 A*24:11	A*24:02 A*30:01	A*24:02 A*24:02	A*02:01 A*33:03

b

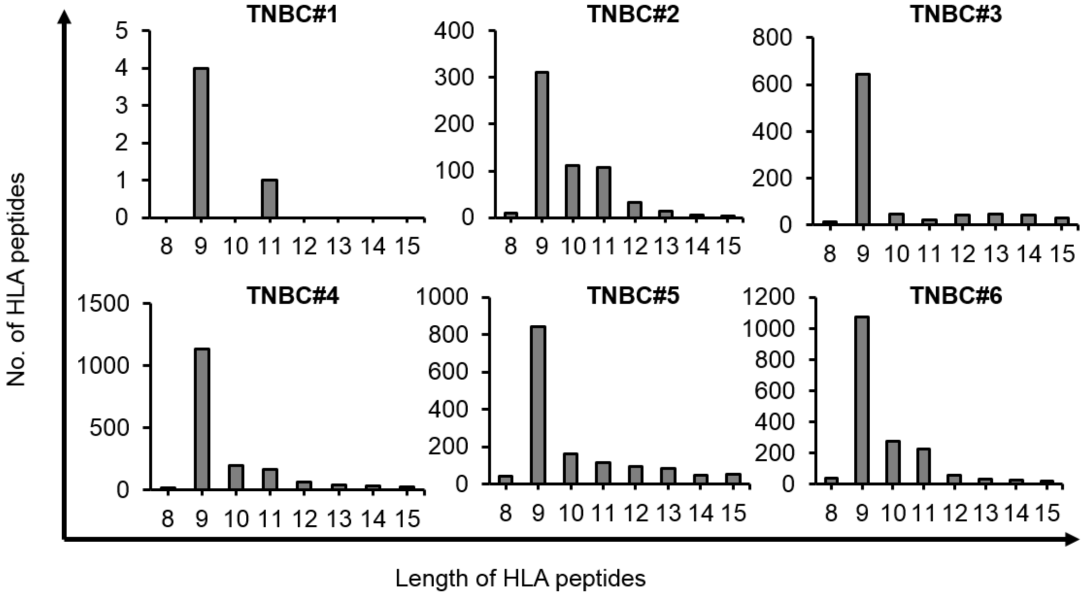

Figure S6. LC-MS/MS identification of eluted peptides with a 1% FDR. (**a**) The number of eluted peptides and the type of HLA-A alleles from six patients with TNBC. (**b**) The typical length of the eluted peptides from individual patients.

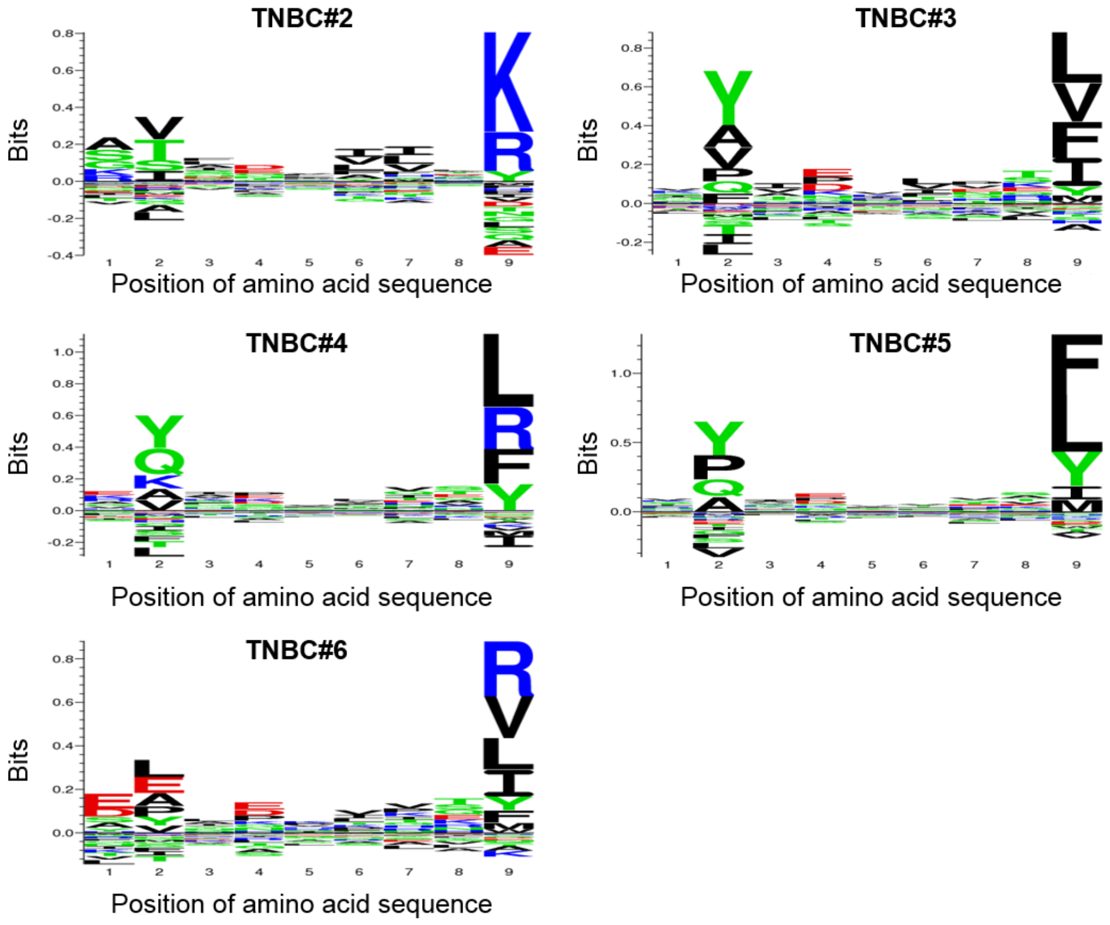

Figure S7. Sequence analysis of the HLA peptides. Logo plots showing sequence analysis of all 9-mer HLA peptides derived from each patient along with amino acid frequency distributions.

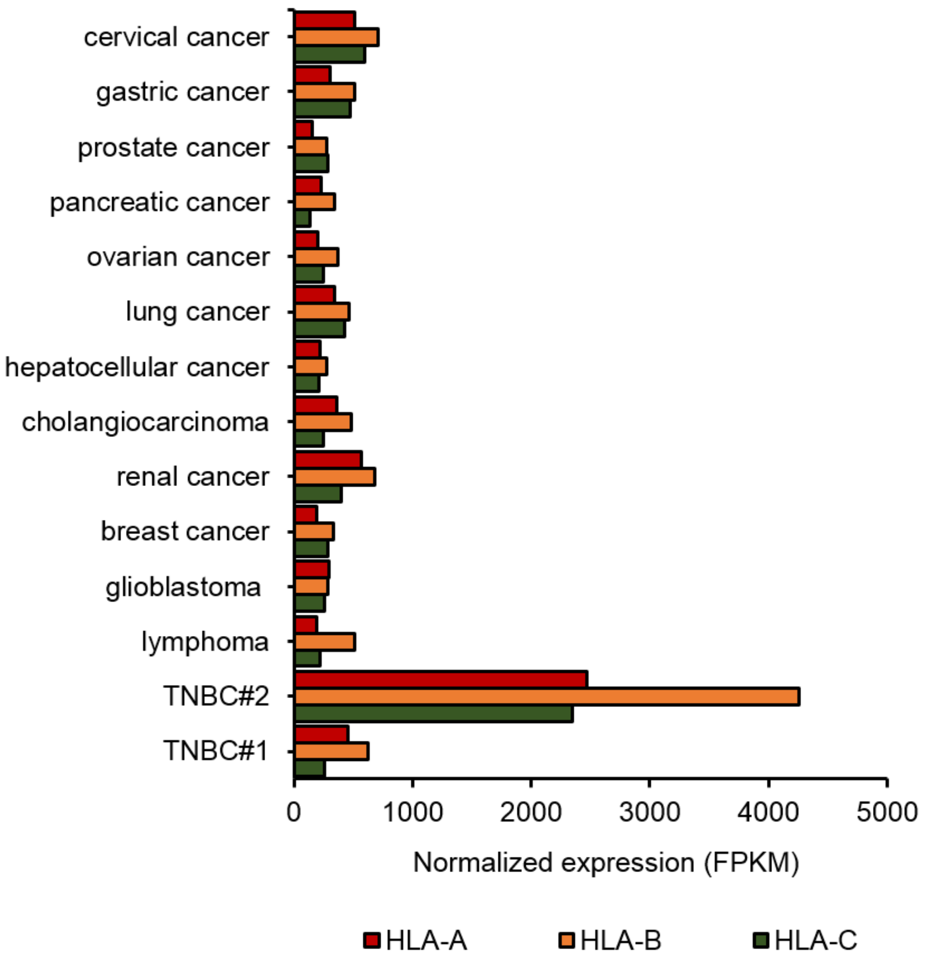

Figure S8. Comparison of expression of three MHC-class-I genes (HLA-A/B/C) in patients TNBC#1 and TNBC#2. Patient TNBC#2 showed stronger expression of MHC-I genes than that in other cancer tissues. The HLA expression of multiple cancers were obtained from the Expression Atlas.

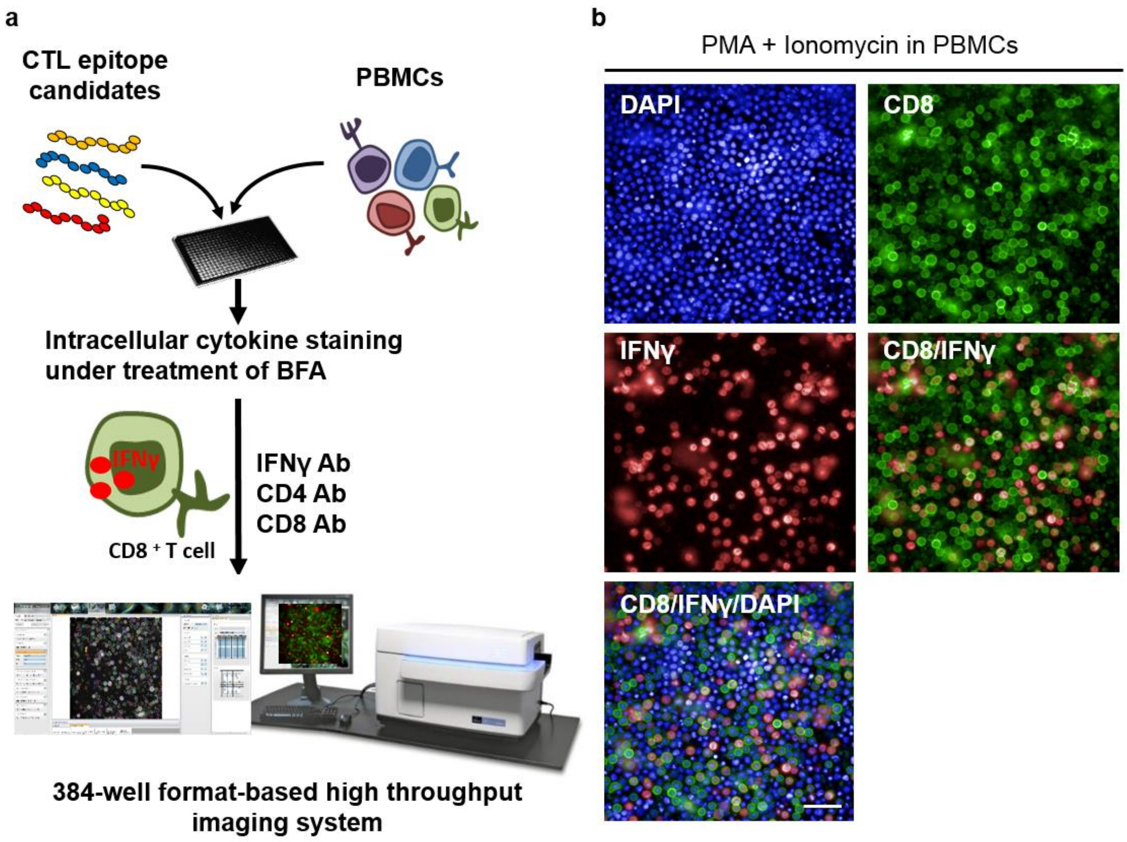

Figure S9. Identification of IFNγ response against peptide-reactive T cells. (**a**) The scheme of the 384-well format-based screening system for detection of IFNγ-producing $CD8^+$ T cells. (**b**) Intracellular IFNγ was detected in PBMCs treated with PMA plus ionomycin. Scale bar, 100 μm.

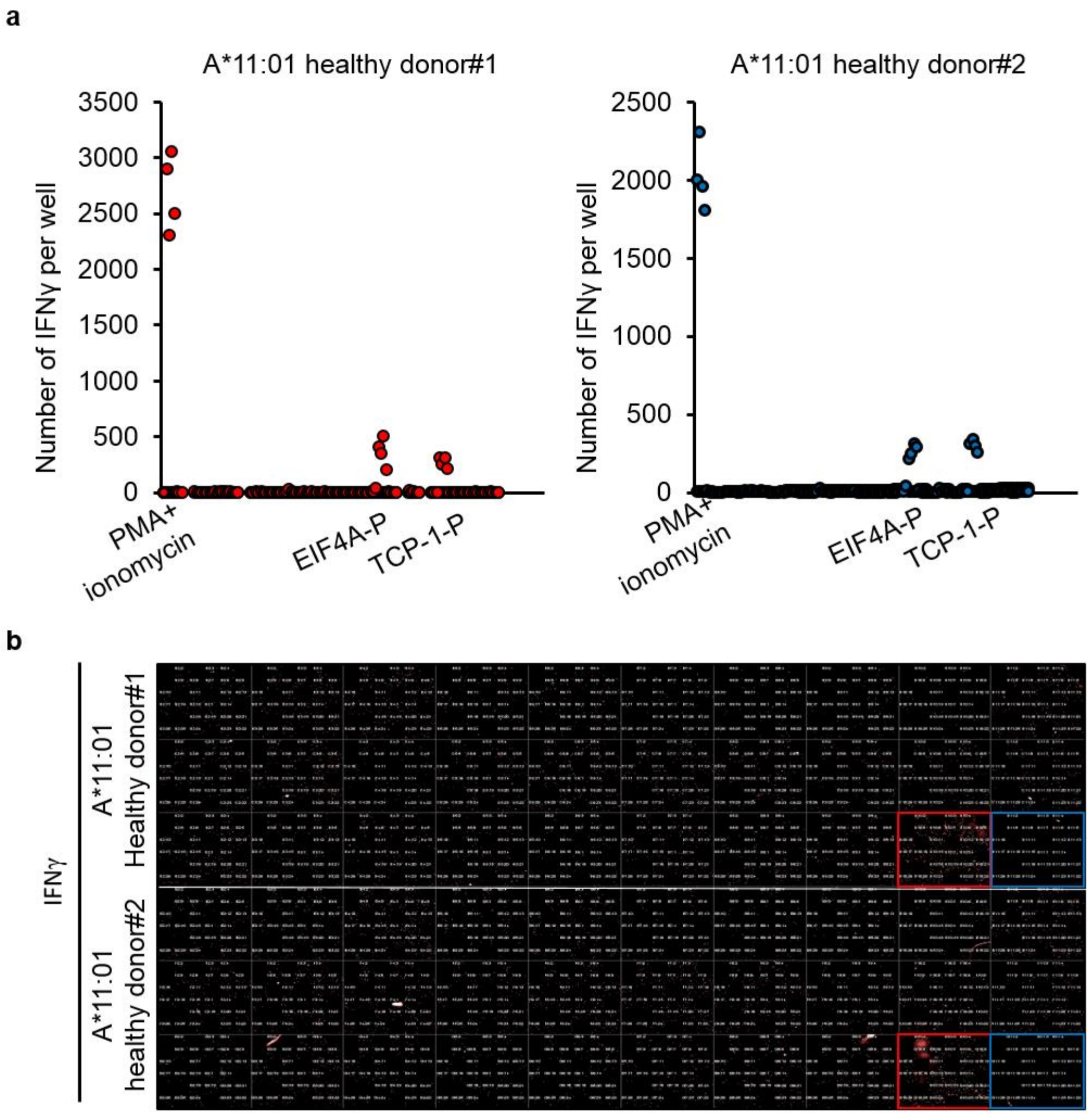

Figure S10. Overview of intracellular IFNγ response of T cells in sample healthy donor #1 and #2. (**a**) Dot plots showing the number of intracellular IFNγ-producing cells per individual wells in the two healthy donors. Quadruple replicate wells were used per treatment. (**b**) The overview images of IFNγ signal per well. Red and blue boxes indicate positive and negative controls, respectively.

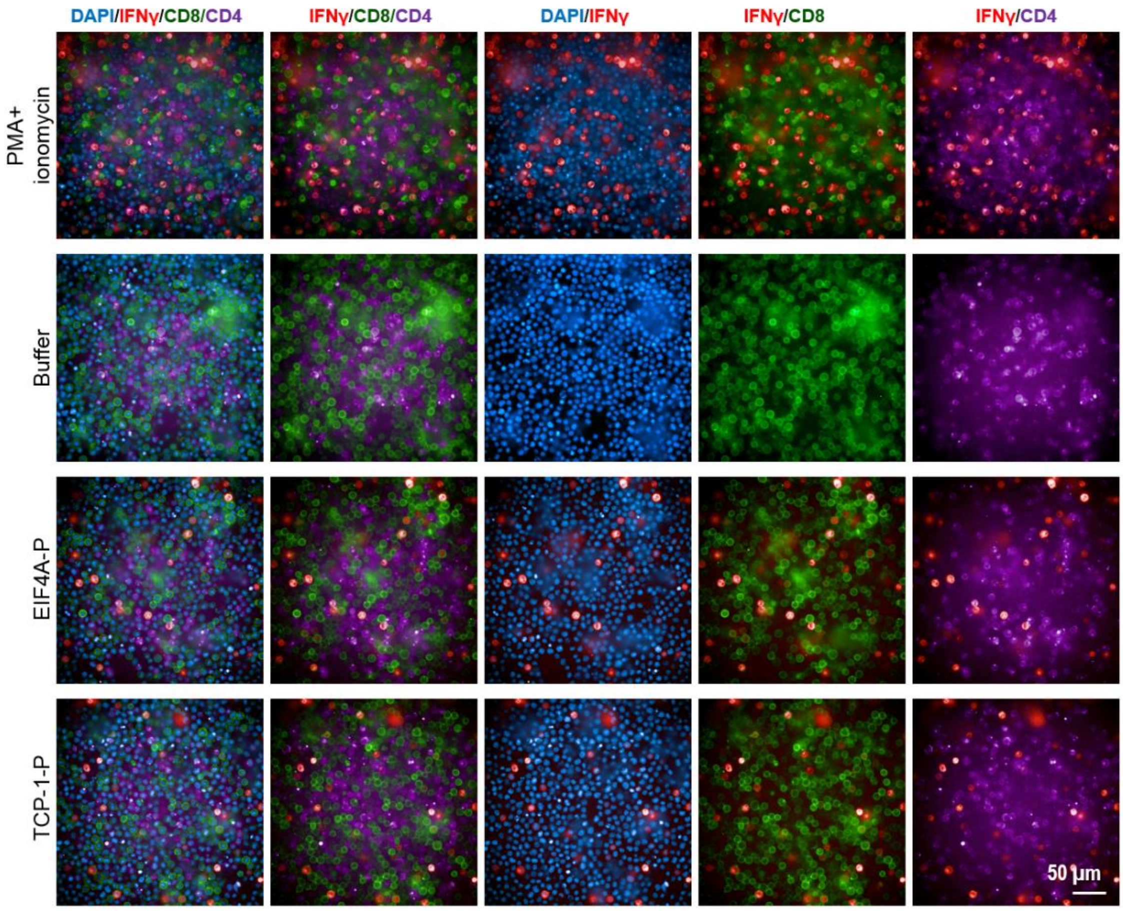

Figure S11. Single-cell imaging of the intracellular IFNγ response. Single-cell-based imaging showing the intracellular IFNγ response of T cells from tissue from healthy donor #1. Fluorescence imaging using the 384-well format was performed after ICS experiments. PMA (50 ng/mL) plus ionomycin (1 µg/mL) was used for positive stimulation in the presence of Brefeldin A treatment to stop the secretion of intracellular IFNγ. Blue, red, green, and violet colors represent nuclear, $CD8^+$ T cell, $CD4^+$ T cell, and intracellular IFNγ staining, respectively. Scale bar, 50 µm.
